# Effect of Reducing Ataxia-Telangiectasia Mutated (ATM) in Experimental Autosomal Dominant Polycystic Kidney Disease

**DOI:** 10.3390/cells10030532

**Published:** 2021-03-03

**Authors:** Jennifer Q. J. Zhang, Sayanthooran Saravanabavan, Gopala K. Rangan

**Affiliations:** 1 Centre for Transplant and Renal Research, Westmead Institute for Medical Research, The University of Sydney, Westmead, NSW 2145, Australia; jennifer.zhang@sydney.edu.au (J.Q.J.Z.); sayan.saravanabavan@sydney.edu.au (S.S.); 2 Department of Renal Medicine, Westmead Hospital, Western Sydney Local Health District, Sydney, NSW 2145, Australia

**Keywords:** autosomal dominant polycystic kidney disease, DNA damage response, ATM, ATR

## Abstract

The DNA damage response (DDR) pathway is upregulated in autosomal dominant polycystic kidney disease (ADPKD) but its functional role is not known. The ataxia-telangiectasia mutated (ATM) and AT and Rad3-related (ATR) protein kinases are key proximal transducers of the DDR. This study hypothesized that reducing either ATM or ATR attenuates kidney cyst formation and growth in experimental ADPKD. In vitro, pharmacological ATM inhibition by AZD0156 reduced three-dimensional cyst growth in MDCK and human ADPKD cells by up to 4.4- and 4.1-fold, respectively. In contrast, the ATR inhibitor, VE-821, reduced in vitro MDCK cyst growth but caused dysplastic changes. In vivo, treatment with AZD0156 by oral gavage for 10 days reduced renal cell proliferation and increased p53 expression in *Pkd1^RC/RC^* mice (a murine genetic ortholog of ADPKD). However, the progression of cystic kidney disease in *Pkd1^RC/RC^* mice was not altered by genetic ablation of ATM from birth, in either heterozygous (*Pkd1^RC/RC^/Atm^+/−^*) or homozygous (*Pkd1^RC/RC^/Atm^−/−^*) mutant mice at 3 months. In conclusion, despite short-term effects on reducing renal cell proliferation, chronic progression was not altered by reducing ATM in vivo, suggesting that this DDR kinase is dispensable for kidney cyst formation in ADPKD.

## 1. Introduction

Autosomal dominant polycystic kidney disease (ADPKD) is the most common hereditary kidney disease [1]. It is due to heterozygous, germ-line mutations, mainly in either *PKD1* or *PKD2*, which encode polycystin 1 and 2, respectively [2]. This causes sporadic formation and growth of multiple kidney cysts and a 50% lifetime risk for kidney failure by age 60 [3,4,5,6]. The loss of polycystin function in distal kidney tubular cells results in an abnormal phenotype characterized by sustained proliferation, evasion of cell death and defective glucose metabolism (Warburg effect) [4,7,8]. Emerging evidence suggests that an increased susceptibility to DNA-damaging events, genomic instability, and dysregulation in DNA damage response (DDR) signaling is also feature of cystic kidney diseases [9,10,11,12,13,14,15]. We previously hypothesized that DNA damage may play a role in ADPKD pathogenesis [16], and demonstrated that the expression of the DDR signaling pathway was dysregulated in both human and early murine ADPKD [17].

Ataxia-telangiectasia mutated (ATM) and AT and Rad3-related (ATR) are phosphoinositide 3-kinase (PI3K)-related kinases (PIKKs) that mediate DDR signaling [18]. Targeting these apical DDR kinases provides opportunities to understand their role in ADPKD pathogenesis, and begin to examine the therapeutic potential for targeting the DDR pathway. In this regard, in a preclinical model of Huntington’s disease (another autosomal dominant genetic condition), the reduction of ATM attenuated behavioral deficits and improved neuropathology [19]. ATM is activated by the MRE11-RAD50-NBS1 (MRN) complex in response to double-stranded DNA breaks and phosphorylates checkpoint kinase-2 (Chk-2) to delay G_1_ to S phase progression (*via* Cdc25A, p53, and p21), allowing time for DNA repair to proceed [20,21,22]. In contrast, ATR and its interacting protein (ATRIP) are recruited for the repair of single-stranded DNA breaks and phosphorylates Chk-1 [22,23]. Our laboratory has previously demonstrated that in human ADPKD, phosphorylated (p) ATM and ATR were localized to cystic kidney epithelial cells, and upregulated transiently in the *Pkd1^RC/RC^* mouse transcriptome on postnatal day 0 [17]. In vitro, constitutively increased p-ATR and p-ATM in human ADPKD kidney tubular cells was associated with enhanced cell survival following exogenous oxidative DNA damage (H_2_O_2_), suggesting DDR upregulation could, in part, promote cystogenesis [17]. Moreover, other studies have demonstrated that knockdown of polycystin-1 in murine inner medullary collecting duct cells and human primary renal epithelial cells caused genomic instability and centrosome amplification, making cells more susceptible to mitotic catastrophe [9]. In this regard, we speculated whether inhibition of either ATR or ATM could further increase genomic instability in cystic kidney epithelial cells, leading to cell death [24].

The functional significance of ATM or ATR in mediating kidney cyst growth in ADPKD is however not known. Therefore, in this study, we hypothesized that reducing either ATM or ATR attenuates cystic kidney disease. To test this hypothesis, we investigated: (i) the efficacy of ATM and ATR kinase inhibitors on cyst growth in vitro; and (ii) the progression of cystic kidney disease in *Pkd1^RC/RC^* mice (genetic ortholog of ADPKD) with heterozygous or homozygous genetic deletion of *Atm* [25]. Our results demonstrate that pharmacological kinase inhibition of ATM reduced cyst growth in vitro and renal cell proliferation in vivo but that the long-term progression of cyst growth in a genetic ortholog of ADPKD was not altered by the reduction or loss of *Atm*. Furthermore, we noted that pharmacological ATR inhibition of ATR was associated with the development of dysplastic changes in cystic kidney epithelial cells in vitro.

## 2. Materials and Methods

### 2.1. Drugs

Kinase inhibitors of ATM (KU-60019, AZD1390, and AZD0156) and ATR (VE-821) were obtained from Selleck Chemicals (Houston, TX, USA). Sirolimus was obtained from LC Laboratories (Woburn, MA, USA). The concentrations and timepoints of the pharmacological inhibitors used in all experiments were based on previous studies [26,27,28,29,30,31,32,33,34].

### 2.2. Cell Lines

Cell lines obtained from the American Type Culture Collection (ATCC, Manassas VA, USA) were used in the experiments: (i) Madin-Darby canine kidney (MDCK) epithelial cells, a normal kidney epithelial cell line derived from *Canis familiaris*; (ii) HK-2, an immortalized proximal tubule cell line derived from normal human kidney cortex (CRL-2190, Lot no. 61218770) [35]; and (iii) ADPKD cell lines: WT 9-7 and WT 9-12, both are immortalized cells derived from a human ADPKD kidney (CRL-2830, Lot no. 58737172 and CRL-2833, Lot no. 60336584). The WT 9-7 and 9-12 cell lines were derived from different cortical cysts from the same female ADPKD patient of unknown age [36]. Both cell lines possess a truncating nonsense variant in *PKD1* (7877C-T) [36,37]. In addition, the 9-7 line has proximal tubule cell characteristics and is heterozygous for the *PKD1* variant, whereas the 9-12 has both proximal and distal tubule cell features and is homozygous for the *PKD1* variant (consisting of a germline and suspected somatic variant), with complete absence of full-length polycystin-1 [36,37]. Cell lines were authenticated by Short Tandem Repeat (STR) DNA profiling. MDCK and HK-2 cells were cultured in a 1:1 ratio of DMEM and F12 and 10% fetal bovine serum (FBS). WT 9-7 and WT 9-12 cells were cultured in DMEM and 10% FBS. All cultures were maintained at 37 °C and 5% CO_2_.

### 2.3. Three-Dimensional (3D) In Vitro Cyst Models

To determine the functional effects of ATM/ATR inhibition on cyst growth, their effects were examined in 3D in vitro models:

MDCK Cyst Model. This is a well-characterized in vitro model that has been widely used to screen the efficacy of novel drugs on kidney cyst growth [38]. MDCK cells were suspended in 0.2 mL of 6 mg/mL ice-cold collagen bovine Type I, supplemented with 10% 10× minimum essential medium (MEM), 10 mM HEPES, 27 mM NaHCO_3_, and 0.2 mM NaOH for neutralization in 24-well plates, as previously described [39]. Plates were incubated at 37 °C for 90 min in a water bath to allow gelation of collagen. To determine the effects of ATM/ATR inhibition, suspended MDCK cells (2 × 10^3^/well) were either untreated or treated with VE-821 or KU-60019 (2, 5, and 10 μM) from Day 0 in the continued presence of forskolin (an agonist of cyclic adenosine monophosphate; 10 μM). The initial experiments, using VE-821 and KU-60019, were performed once with *n* = 3 wells per treatment group with forskolin only as control, and cyst growth was quantified on Days 6 and 12. Subsequent further studies in suspended MDCK cells (2 × 10^4^/well) were then performed using other ATM inhibitors. In these further studies, cells were treated either with the ATM inhibitors, AZD0156 (0.2, 0.5, or 1 μM) or AZD1390 (0.2, 0.5, or 1 μM), from Day 0 in the continued presence of forskolin, and cyst growth was compared to groups treated with vehicle (0.1% ethanol) and sirolimus (0.05 μM) (a classical agent that potently reduces kidney cyst growth) [40] on Days 4 and 8. Experiments were performed twice with *n* = 3 wells per treatment group (*n* = 6 total). Media with treatments was replaced every 48 h.

Human ADPKD Cyst Model (WT 9-12). To further evaluate the efficacy of DDR inhibitors, their effect in a 3D in vitro cyst model using human ADPKD cells was also evaluated. In contrast to the MDCK cyst model, human-derived cells have been less widely utilized in previous studies. In the present study, the protocol for human cyst culture was adapted from previous report [41], with modifications. WT 9-12 cells (2 × 10^4^ cells/well) were suspended in Matrigel^®^ Growth Factor Reduced (Corning, Corning NY, USA) in a 96-well plate. The plate was incubated for 15 min at 37 °C to promote gelation. Following this, 200 μL of DMEM supplemented with 20% FBS, 25 ng/mL human epidermal growth factor, 5 μg/m/L hydrocortisone, and 1X ITS Liquid Media Supplement was added (Sigma-Aldrich, St Louis MO, USA). Suspended WT 9-12 cells were treated with either vehicle (0.1% ethanol) or AZD0156 (0.2, 0.5 or 1 μM) from day 0 in the continued presence of forskolin. Cyst formation was observed on days 6, 12, and 18. Experiments were performed once with *n* = 6 wells per treatment group. Media with treatments was replaced every 48 h.

Quantification of Cyst Growth. To quantify cyst growth in response to treatments, between 10 and 12 images of individual cysts, chosen at random, were obtained at 10× magnification from each well at each timepoint. Individual cyst diameter was measured using ImageJ software (v1.47; National Institutes of Health, Bethesda, MD, USA). These measurements were averaged to provide a mean value for cyst diameter for each well.

### 2.4. Assessment of Cell Viability, Cytotoxicity, and Proliferation

For all assays, MDCK, HK-2, WT 9-7, and/or WT 9-12 cells were seeded in 96-well plates (5 × 10^3^ cells/well) and cultured for 24 h prior to treatment. For cell viability assays, cells were either untreated or treated with VE-821 or KU-60019 (2, 5, and 10 μM) for 24 h. The MTT assay is an indicator of the number of metabolically active cells and is therefore a measure of cell viability, proliferation, and cytotoxicity [42]. The MTT assay (11465007001, Roche Diagnostics, Mannheim, Germany) was performed according to manufacturer’s instructions. Absorbance was measured at 570 nm (reference 750 nm). Cell viability was calculated by 100× (Absorbance of Sample/Average Absorbance of Untreated). MTT experiments were performed once with *n* = 3 wells per treatment group in each experiment.

To further clarify effects on cell cytotoxicity in response to AZD0156 cells were treated with vehicle (0.1% ethanol) or AZD0156 (0.2, 0.5 or 1 μM) for 24, 48, or 72 h, and the lactate dehydrogenase (LDH) assay (CytoTox 96^®^ Non-Radioactive Cytotoxicity Assay, G1780; Promega, Madison WI, USA) was performed according to manufacturer’s instructions with absorbance measured at 490 nm. Cell cytotoxicity was calculated by 100× (Absorbance of Sample/Average Absorbance of Maximum LDH Release Control). To determine cell proliferation in response to AZD0156, the bromodeoxyuridine (BrdU) assay (Cell Proliferation ELISA, BrdU (colorimetric), 11647229001; Roche) was performed according to manufacturer’s instructions. Absorbance was measured at 450 nm (reference 690 nm). Cell proliferation was calculated by 100× (Absorbance of Sample/Average Absorbance of Vehicle). LDH and BrdU experiments were performed twice with *n* = 4 wells per treatment group in each experiment (*n* = 8 total).

### 2.5. Mice

*Pkd1^RC/RC^ C57BL/6J*. *Pkd1^RC/RC^* mice are an inbred knock-in of a *PKD1* hypo-morphic allele (*PKD1 p.R3277C*), described in detail elsewhere [43]. *Atm^tm1Awb^ B6.129S6J. Atm^+/−^* mice were obtained from The Jackson Laboratory (#008536; Bar Harbor, ME, USA) in July 2019 and used to establish a breeding colony at Australian Bio-Resources (Moss Vale, NSW Australia) [25,44]. *Atm^+/−^* mice were generated by disrupting a 179-base pair (bp) exon with a PGK*neo* gene at nucleotide position 5790 in the opposite orientation to *Atm* transcription, introducing a truncating mutation [25]. *Atm^+/−^* mice are viable, fertile, and breed with expected Mendelian ratios; have no phenotype; and are comparable in size to wild-type up to 8 months [25]. In contrast, *Atm^−/−^* mice exhibit growth retardation, neurologic dysfunction, infertility, immunodeficiency, malignancies, chromosomal instabilities, and sensitivity to ionizing radiation [25].

Maintenance, Housing, and Animal Ethics. Mouse colonies were maintained at Australian BioResources and then transferred and housed at the Biological Services Facility at the Westmead Institute for Medical Research (Westmead, NSW, Australia) for experimental studies. Mice received food and water *ad libitum* and were housed under standard conditions (temperature: 21 ± 2 °C; humidity: 55 ± 15%; artificial lighting; light: dark cycle 1900–0700). All procedures for the animal studies were approved by the Western Sydney Local Health District Animal Ethics Committee (Protocol numbers 5134 and 5157).

### 2.6. In Vivo Mouse Experiments

Two experiments were performed to investigate the in vivo effects of reducing ATM. In the first study, to determine the effect of pharmacological ATM kinase inhibition, groups of *Pkd1^RC/RC^* mice (*n* = 4 per group; aged 2–3 months) were randomized to receive either: (i) vehicle (0.5% (*w*/*v*) hydroxypropyl methylcellulose (HPMC) and 0.1% (*v*/*v*) Tween 80 in ultrapure water), (ii) low dose AZD0156 (5 mg/kg body weight/day), or (iii) high dose AZD0156 (20 mg/kg body weight/day). Mice were dosed via oral gavage on Monday, Wednesday, and Friday for 2 consecutive weeks for a total study duration of 10 days. Mice were sacrificed 4 h after dosing on day 10. The vehicle composition, drug dose, and frequency of administration were based on previous studies [29,30,34].

In the second study, to determine the effect of genetic ablation of ATM on the long-term progression of cystic kidney disease, groups of wild-type (*Pkd1^+/+^*) and *Pkd1^RC/RC^* mice with either *Atm^+/+^*, *Atm^+/−^*, or *Atm^−/−^* were sacrificed at 3 months of age. A two-step process was used to produce compound mice that were homozygous *Pkd1* hypo-morphic allele (*Pkd1 p.R3277C*) [43] together with heterozygosity for *Atm*. First, female *Pkd1^RC/RC^*/*Atm^+/+^* and male *Pkd1^+/+^*/*Atm ^+/−^* were mated, and breeder pairs from the progeny with the correct genotype (*Pkd1^+/RC^/Atm^+/−^*) were formed to produce mice of the following genotypes: (i) *Pkd1^+/+^*/*Atm^+/+^* (*n* = 4 per gender), (ii) *Pkd1^+/+^*/*Atm^+/−^* (*n* = 4 per gender), (iii) *Pkd1^+/+^*/*Atm^−/−^* (*n* = 2 male, *n* = 1 female), (iv) *Pkd1^RC/RC^*/*Atm^+/+^* (*n* = 10 per gender), (v) *Pkd1^RC/RC^*/*Atm^+/−^* (*n* = 10 per gender), and (vi) *Pkd1^RC/RC^*/*Atm^−/−^* (*n* = 8 male, *n* = 2 female). In addition, to evaluate the effects of ATM deficiency on a longer period of follow-up, a subset of *Pkd1^RC/RC^/Atm^+/−^* mice (*n* = 6 male) were sacrificed at 6 months of age and compared to archival control samples from a previous study (*Pkd1^RC/RC^*/*Atm^+/+^*; *n* = 6 male) [17]. The timepoints chosen for follow-up were based on disease progression described by previous studies [17,43,45,46]. Genotyping was undertaken using ear-punch DNA with a two-step PCR reaction performed by Garvan Molecular Genetics (Garvan Institute for Medical Research, Sydney, NSW, Australia; Figure S1). At the completion of follow-up in both studies, mice were anaesthetized by an intraperitoneal injection of 20% ketamine: 10% xylazine, and blood was collected via cardiac puncture, as previously described [15]. A mid-line laparotomy was performed, and the aorta and inferior vena cava were transected. The kidney and hearts were removed, and either immediately snap-frozen in liquid nitrogen or immersion-fixed [10% neutral buffered formalin (NBF) or methyl Carnoy’s] for 24 h and then embedded in paraffin for histological analysis.

### 2.7. Histology, Immunohistochemistry, and Histological Quantification

Kidney tissue sections (4 µm in thickness) were cut and stained with Periodic Acid Schiff (PAS) and 0.1% Sirius Red/0.1% Fast Green in picric acid. Antigen retrieval was performed by Decloaking Chamber^TM^ NxGen (95 °C for 40 min; Biocare Medical; Pacheco, CA, USA) in 1X Antigen Decloaker (Biocare Medical). Sections, blocked with Background Sniper (Biocare Medical), were incubated overnight at 4 °C with primary antibodies. Primary antibodies used were: (i) Ki-67 (1:200, MA5-14520; Invitrogen), (ii) cleaved caspase-3 (Asp175) (1:400, 9661; Cell Signaling Technology, Danvers, MA, USA), and (iii) anti-phospho-histone H2A.X (Ser139) (1:480, 9718; Cell Signaling Technology). Secondary biotinylated antibodies (1:200 dilution) were applied for 30 min at room temperature, followed by Vectastain ABC reagent (Vector Laboratories, Burlingame, CA, USA) for 20 min and diaminobenzidine and counterstained with methyl green.

For quantification, whole-slide digital images were acquired using a slide scanner (NanoZoomer v1, Hamamatsu Photonics, Japan) and whole-slide analysis was performed using the positive pixel algorithm on Aperio ImageScope (v11.2.0.780, Leica Biosystems, Wetzlar, Germany). For percentage cystic area from PAS slides, the perimeters of the sections from digitally acquired images were outlined using the *positive selection pen*, while excluding the medullary regions. The white and colored areas of the image were derived from the positive pixel count algorithm. For percentage-positive pixels in images, a total of eight 20× fields of view from the kidney cortex were analyzed and averaged for each mouse.

### 2.8. Western Blot of Renal p53

Protein lysates were prepared using radioimmunoprecipitation assay (RIPA) buffer with protease (P8340; Sigma-Aldrich) and phosphatase inhibitors (4906845001; Roche, Basel, Switzerland). Further, 65 µg of protein was electrophoresed on a 4–15% Mini-PROTEAN^®^ TGX Stain-Free^TM^ precast gel (Bio-Rad Laboratories, Hercules, CA, USA) and transferred to a 0.45 µm PVDF membrane (Bio-Rad Laboratories) by wet transfer. The membrane was then blocked (5% bovine serum albumin (BSA) diluted in Tris-buffered saline with 0.1% Tween (TBST)) for 1 h, followed by overnight incubation at 4 °C with the primary antibody (p53 (1:1000, 2524; Cell Signaling Technology)). The membrane was then incubated with the secondary antibody (infrared fluorescent-conjugate) for 1 h at room temperature. Blots were scanned on the Odyssey Imaging System (LI-COR Biosciences, Lincoln, NE, USA). Densitometry was quantified using Image Studio Lite (v5.2.5, LI-COR Biosciences) and ImageLab^TM^ Software (v6.0.1, Bio-Rad Laboratories) and normalized against total protein through stain-free imaging technology (Bio-Rad Laboratories) [47,48].

### 2.9. Statistics

All statistical analyses were performed using JMP^®^ Pro (v14.2.0, SAS Institute, Cary, NC, USA). Data were reported as means ± standard deviation (SD). Where appropriate, differences between groups were determined by 2-sample Mann–Whitney U test, 2-sample independent *t*-test, or one-way analysis of variance (ANOVA), followed by post hoc analysis with the Tukey–Kramer HSD test. Significance was defined as *p* < 0.05. The sample size for animal studies was determined based on kidney weight variation from a historical dataset of *Pkd1^RC/RC^* mice [17] and with consideration of the combined BL/6 and 129 background of the *ATM^−/−^* mice, as described previously [49].

## 3. Results

### 3.1. Pharmacological Inhibition of ATR or ATM Reduces Cyst Growth In Vitro

#### 3.1.1. Effect of VE-821 and KU-60019 on MDCK Cyst Model

**Figure 1:**
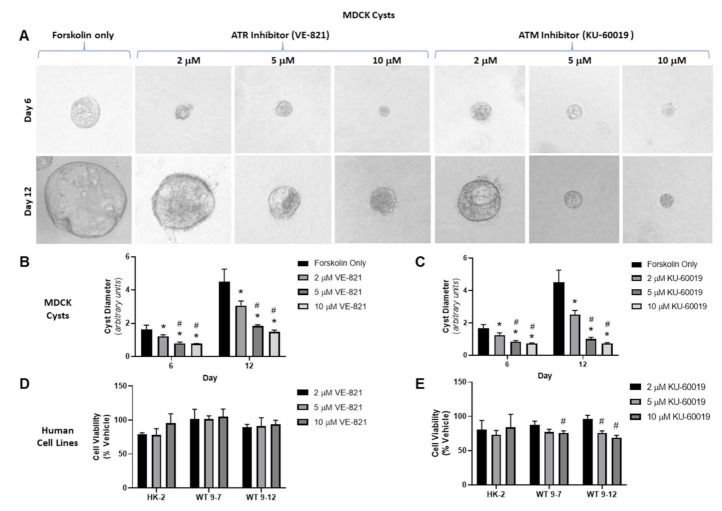
Effect of VE-821 (ATR inhibitor) and KU-60019 (ATM inhibitor) on three-dimensional (3D) Madin-Darby canine kidney (MDCK) cyst growth and cell viability (MTT assay) in human kidney tubular cell lines. (**A**) Representative images of MDCK cysts treated with forskolin alone, VE-821 (2, 5, and 10 µM) or KU-60019 (2, 5, and 10 µM) on Days 6 (upper panel) and 12 (lower panel) (Images were obtained using 10x objective). (**B**) and (**C**) Mean cyst diameter of MDCK cysts on Days 6 and 12, treated with either VE-821 or KU-60019 as determined by image analysis; (**D**) and (**E**) Mean number of viable cells (as a percentage of vehicle, measured by the MTT assay) in HK-2, WT 9-7, and WT 9-12 cells treated with either VE-821 (2, 5, and 10 µM, (**D**)) or KU-6001 (2, 5, and 10 µM, (**E**)) for 24 h. * *p* < 0.05 compared to forskolin alone, # *p* < 0.05 compared to 2 µM by one-way ANOVA, followed by post hoc analysis with the Tukey–Kramer HSD test. Data presented as means ± SD (*n* = 3 per group).

Initial experiments to evaluate the efficacy of ATR and ATM inhibition were performed using VE-821 (IC_50_, 26 nM) and KU-60019 (IC_50_, 6.3 nM). In the MDCK cyst model, both VE-821 and KU-60019, reduced cyst diameter in a dose-dependent manner by up to 3.0- and 6.1-fold, respectively (Figure 1A–C). Unexpectedly, VE-821 treatment also produced cysts with a higher percentage of “hair”-like protrusions, suggesting dysplastic change (Figure S2 and Table S1). To investigate the mechanisms underlying the reduction in cyst growth, we also investigated the effects of VE-821 and KU-60019 on cell proliferation and cytotoxicity. As shown in Table 1, both VE-821 and KU-60019 reduced BrdU incorporation in MDCK monolayers in a dose-dependent manner. Furthermore, neither compound was directly cytotoxic to MDCK cells, as determined by LDH leakage (Table 1). KU-60019 caused a small but significant decrease in percentage LDH leakage compared to vehicle probably due to a reduction in cell number (Table 1). Finally, we also evaluated whether ATM/ATR kinase inhibition had selective effects in human kidney cells mutated for *PKD1* (WT 9-7 and WT 9-12 cells) compared to normal (HK-2 cells). As shown in Figure 1D, the effect of VE-821 on the number of viable cells (as determined by the MTT assay) was similar in all three human kidney cell lines. In contrast, KU-60019 reduced the number of viable cells at higher doses (5–10 mM) in ADPKD cells compared to normal human kidney cells (Figure 1E). Due to the possible dysplastic change on cysts observed with VE-821 and also the possibility that KU-60019 may have more selective effects on *PKD1*-mutated cells, subsequent studies focused on the effect of inhibiting ATM.

#### 3.1.2. Effect of AZD1390 and AZD0156 on MDCK Cyst Model

**Figure 2:**
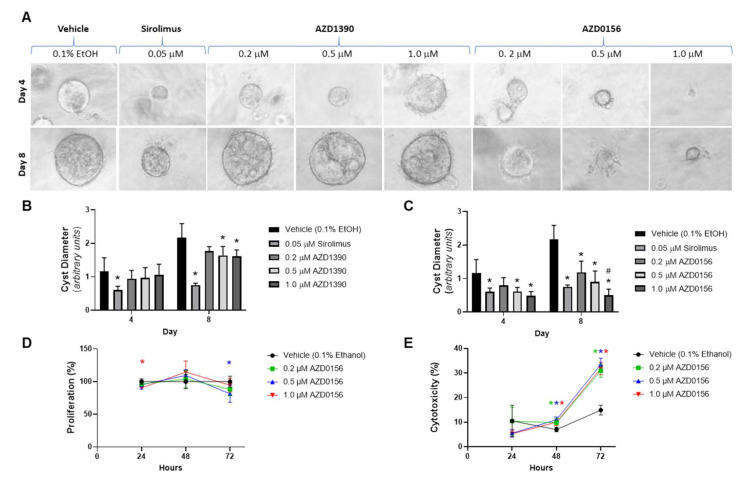
Effect of ataxia-telangiectasia mutated (ATM) inhibitors (AZD1390 and AZD0156) on MDCK cyst growth, cell proliferation and cytotoxicity in vitro. MDCK cysts were treated with either vehicle (0.1% ethanol (EtOH), sirolimus (0.05 μM), AZD1390 (0.2, 0.5, and 1 μM) or AZD0156 (0.2, 0.5, and 1 μM) for up to 8 days. (**A**) Representative images of MDCK cysts treated with either vehicle, sirolimus, AZD1390 or AZD0156 on Days 4 (upper panel) and 8 (lower panel) (Images were obtained using 10× objective). (**B**) and (**C**) Mean cyst diameter in response to AZD1390 (**B**) and AZD0156 (**C**) on Days 4 and 8. * *p* < 0.05 compared to vehicle, and # *p* < 0.05 compared to 0.2 µM of respective treatment. (**D**) Mean cell proliferation (measured by the BrdU assay) and (**E**) Mean percentage cytotoxicity (measured using the LDH assay) following treatment with either AZD0156 or vehicle. * *p* < 0.05 compared to vehicle (color of asterix corresponds to the respective line color). All *p*-values in figure were determined by one-way ANOVA, followed by post hoc analysis with the Tukey–Kramer HSD test, and data presented as means ± SD (*n* = 6 per group).

To further verify the effect of ATM inhibition on in vitro cyst growth and also to select candidates for in vivo testing, we next evaluated the efficacy of two orally bioavailable analogues: (i) AZD1390 (IC_50_, 0.78 nM, >10,000-fold selectivity for ATM) [29], and (ii) AZD0156 (IC_50_ 0.58 nM, >1000-fold selectivity for ATM) [30]. Both ATM inhibitors are in Phase I clinical trials, whereas KU-60019 is limited to in vitro studies [50]. As shown in Figure 2, AZD1390 only reduced MDCK cyst growth on Day 8 at a concentration of 1 μM (by 1.4-fold), whereas AZD0156 was more potent, decreasing cyst diameter by up to 4.4-fold and with comparable efficacy to sirolimus (Figure 2A–C). Interestingly, unlike KU-60019, AZD0156 did not alter BrdU synthesis but rather increased percentage LDH leakage in MDCK cell monolayers compared to vehicle (Figure 2D,E).

#### 3.1.3. Effect of AZD0156 on Human ADPKD Cysts

**Figure 3:**
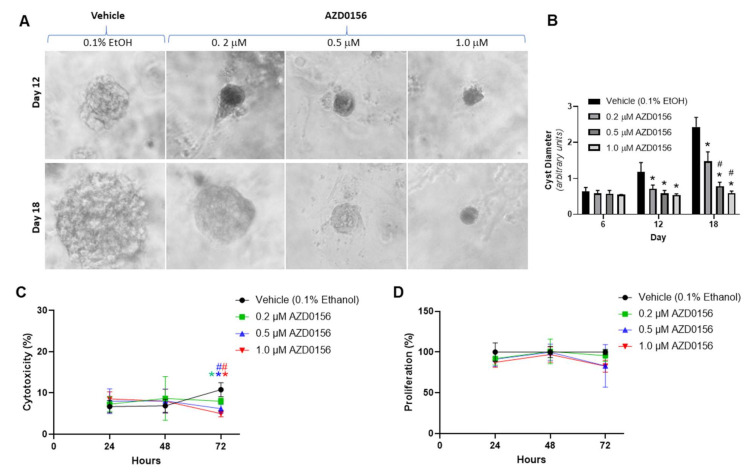
Effect of AZD0156 (ATM inhibitor) on in vitro *cyst* growth in human ADPKD cells. Human ADPKD cysts derived from WT 9-12 cells were treated with either vehicle [0.1% ethanol (EtOH)] or AZD0156 (0.2, 0.5, and 1 μM) for 18 days. (**A**) Representative images of human ADPKD cysts treated with AZD0156 on Days 12 (upper panel) and 18 (lower panel) (Images were obtained using 10x objective). (**B**) Mean Cyst diameter on Days 6, 12, and 18 in the experimental groups. * *p* < 0.05 compared to vehicle, and # *p* < 0.05 compared to 0.2 µM AZD0156. (**C**) Mean percentage cytotoxicity (measured using the LDH assay) and (**D**) Mean cell proliferation (measured by the BrdU assay) following treatment with either AZD0156 or vehicle. * *p* < 0.05 compared to vehicle and # *p* < 0.05 compared to 0.2 µM AZD0156 (color of asterix/hash corresponds to the respective line color). All *p*-values in figure were determined by one-way ANOVA, followed by post hoc analysis with the Tukey–Kramer HSD test, and data presented as means ± SD (*n* = 6 per group).

As MDCK cells do not have mutations in PKD-causative genes, we further evaluated the effect of ATM inhibition in a human ADPKD cyst model using WT 9-12 cells, which possess a homozygous *PKD1* variant [36,37,41]. In this model, the formation of cysts was slower compared to those derived from MDCK cells (requiring 18 days to reach maximal size) and a higher proportion of cysts had an eccentric shape (Figure 3A). Consistent with MDCK cysts, AZD0156 also reduced cyst growth in a dose-dependent manner by up to 4.1-fold by day 18 in WT 9-12-derived cysts (Figure 3B). Furthermore, as shown in Figure 3C,D, AZD0156 did not alter cell proliferation and reduced percentage LDH leakage in WT 9-12 monolayers.

### 3.2. AZD0156 Reduces Renal Cell Proliferation and Increases p53 in Pkd1^RC/RC^ Mice

**Figure 4:**
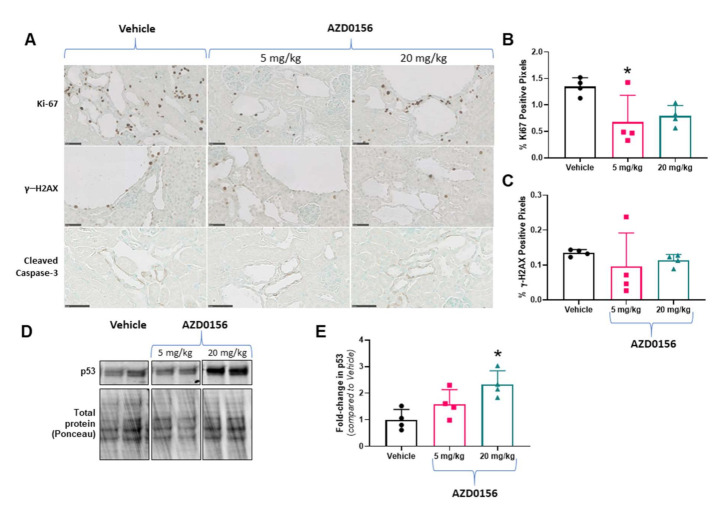
Effect of AZD0156 on renal cell proliferation, DNA damage and p53 in *Pkd1^RC/RC^* mice. (**A**) Representative histological images of renal cortex showing Ki-67+ cells (DAB-positive cells) (upper panel), gH2AX (middle panel), and cleaved caspase-3 (lower panel) in the experimental groups. Scale bars, 50 µM. (**B**) Mean Ki-67-positive pixel quantification in the experimental groups. (**C**) Mean γ-H2AX-positive pixel quantification in the experimental groups. (**D**,**E**) Representative western blots and quantitative analysis for renal p53 expression; * *p* < 0.05 compared to vehicle; Data presented as means ± SD (*n* = 4 per group).

**Figure 5:**
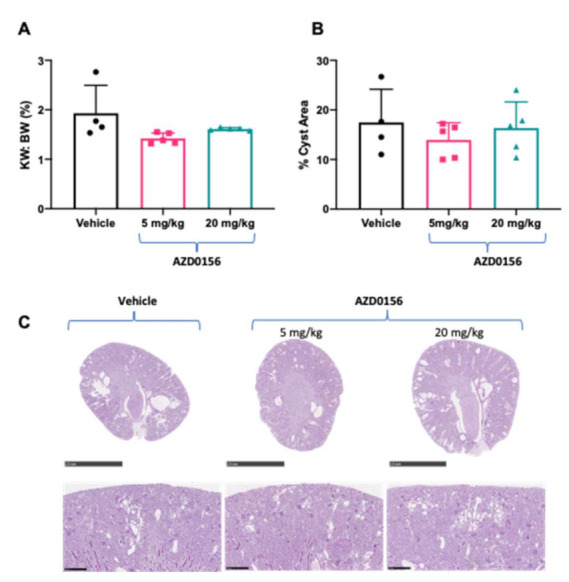
Effects of AZD0156 (5 or 20 mg/kg/day) via oral gavage for 10 days on body weight, kidney enlargement and cyst area in *Pkd1^RC/RC^* mice. (**A**) Mean kidney weight to body weight ratio (KW: BW) in the experimental groups. (**B**) Mean percentage cyst area in experimental groups. (**C**) Representative whole-slide digital images of Periodic Acid Schiff (PAS)-stained kidney sections from mice in the experimental groups. Scale bars in top panel indicates 2.5 mm and bottom panel 0.25 mm. Data presented as means ± SD (*n* = 4–5 per group).

We next evaluated the short-term in vivo effect of AZD0156 on biomarkers of cystic kidney disease in *Pkd1^RC/RC^* mice. Treatment with AZD0156 for a total of 10 days had no adverse effects on general health or body weight in *Pkd1^RC/RC^* mice (change in body weight from day 0 to 10: vehicle: 0.18 ± 0.63, AZD0156 5 mg/kg: −0.38 ± 0.48, and AZD0156: 20 mg/kg: −0.18 ± 0.53 g; *p* > 0.05). In *Pkd1^RC/RC^* mice, AZD0156 reduced renal cell proliferation, measured by Ki-67, compared to vehicle (Figure 4A,B). In contrast, although the DNA damage marker γ-H2AX was not different between treatment groups (Figure 4A,C), AZD0156 increased renal p53 (Figure 4D,E). Due to the essential role of ATM in apoptosis [22], cleaved caspase-3 (Asp175) was also investigated. Cleaved-caspase-3 was expressed in distal tubules but was not altered by AZD0156 (Figure 4A). Furthermore, due to the short duration of this study, as expected, no changes in kidney enlargement and percentage cyst area were observed following AZD0156 treatment (Figure 5).

### 3.3. Progression of Cystic Kidney Disease Is Not Altered in Pkd1^RC/RC^/Atm^+/−^ Mice

**Figure 6:**
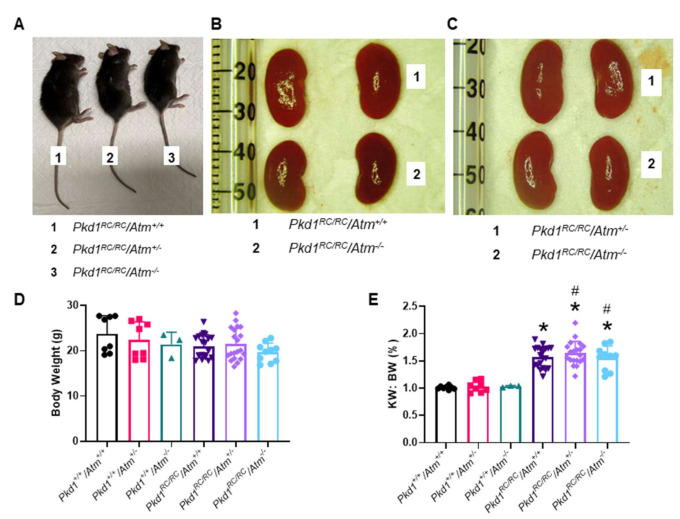
Effect of ATM reduction on the progression of cystic kidney disease in *Pkd1^RC/RC^* mice at 3 months of age. (**A**) Representative macroscopic appearance of male *Pkd1^RC/RC^* mice either with *Atm^+/+^* (wild-type), *Atm*^+/−^ (heterozygous), or *Atm^−/−^* (homozygous) genotype, and this was similar in all experimental groups. (**B**) and (**C**) Representative images of *Pkd1^RC/RC^* mouse kidneys with either *Atm^+/+^* or *Atm^−/−^* genotype. (**D**) Mean body weight of the experimental groups. (**E**) Mean two kidney weight to body weight ratio (KW: BW) in the experimental groups. * *p* < 0.05 compared to wild-type mice (*Pkd1^+/+^/Atm^+/+^*) and # *p* < 0.05 compared to *Atm* genotype matched *Pkd1^+/+^*. Data presented as means ± SD (*n* = 8 per wild-type group; *n* = 4 males and *n* = 4 females; *n* = 20 per *Pkd1^RC/RC^* mouse group; *n* = 10 males and *n* = 10 females).

**Figure 7:**
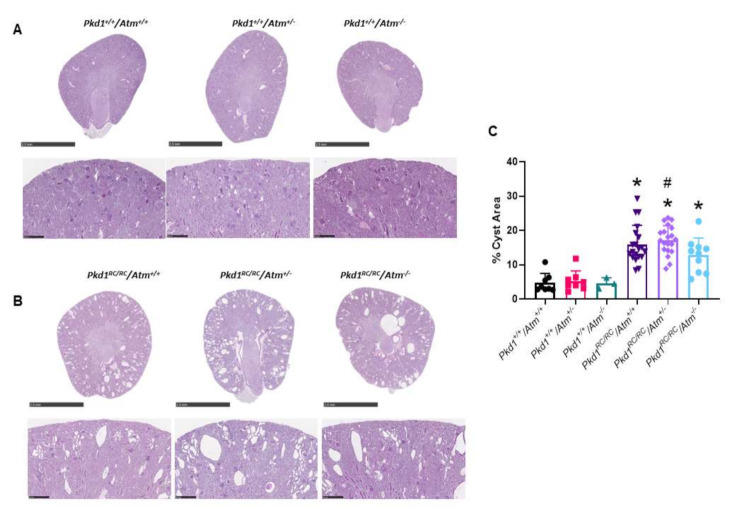
Effect of loss of ATM on renal histology in *Pkd1^RC/RC^* mice at 3 months of age. (**A**,**B**) Representative whole-slide digital images of Periodic Acid Schiff (PAS)-stained kidney sections from male wild-type (**A**) *Pkd1^RC/RC^* (**B**) mice. Scale bars in top panel indicates 2.5 mm and bottom panel 0.25 mm. (**C**) Mean percentage cyst area in the experimental groups calculated as the percentage of white area (no stain) in the total cortical area. * *p* < 0.05 compared to wild-type mice (*Pkd1^+/+^/Atm^+/+^*) and # *p* < 0.05 compared to *Atm* genotype matched *Pkd1^+/+^*. Data presented as means ± SD (*n* = 8 per wild-type group; *n* = 4 males and *n* = 4 females; *n* = 20 per *Pkd1^RC/RC^* mouse group; *n* = 10 males and *n* = 10 females).

**Figure 8:**
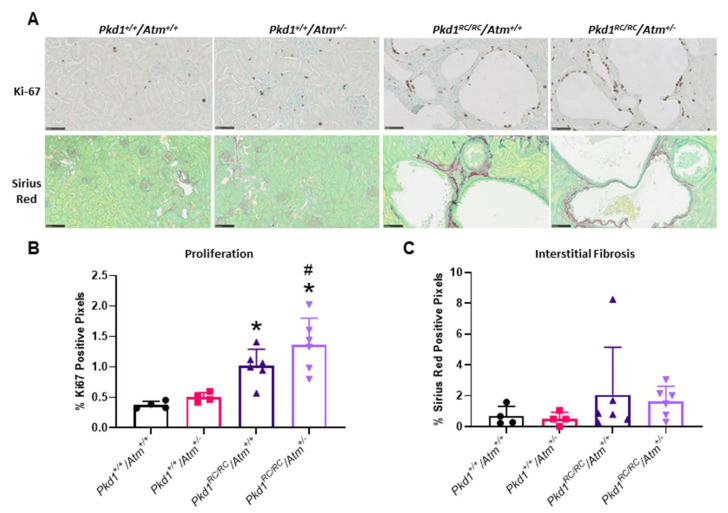
Effect of loss of ATM on renal fibrosis and proliferation in *Pkd1^RC/RC^* mice at 3 months of age. (**A**) Representative histological images of Sirius Red deposition (upper panel) and Ki-67-positive staining (lower panel) in the experimental groups. Scale bars, 50 µM. (**B**) Mean renal fibrosis score calculated by Sirius Red-positive staining. (**C**) Mean renal cell proliferation, as determined by quantification of Ki-67-positive staining. * *p* < 0.05 compared to wild-type mice (*Pkd1^+/+^/Atm^+/+^*) and # *p* < 0.05 compared to *Atm* genotype matched *Pkd1^+/+^*. Data presented as means ± SD (*n* = 4–6 per group).

**Figure 9:**
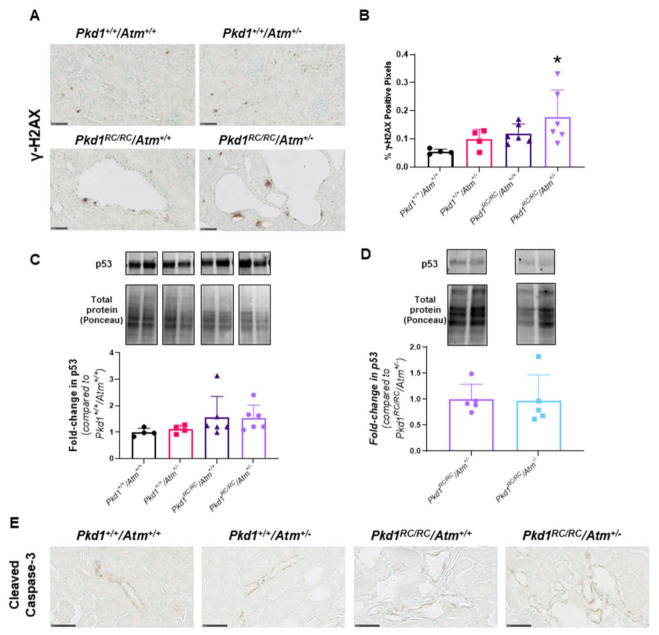
Effect of loss of ATM on DNA damage and p53 expression. (**A**) Representative histological images of γ-H2AX-positive staining in the renal cortex in the experimental groups. Scale bars, 50 µM. (**B**) Mean γ-H2AX-positive staining score in the experimental groups. * *p* < 0.05 compared to *Pkd1^+/+^/Atm^+/+^*. (**C**) Representative Western blot and quantification of renal p53 protein in the experimental groups. (**D**) Representative Western blots and quantification of p53 in *Pkd1^RC/RC^* mouse kidneys with *Atm^+/−^* or *Atm*^−/−^ as fold-change compared to *Pkd1^RC/RC^/Atm^+/−^*. Data presented as mean ± SD (*n* = 4–6 per group). (**E**) Representative histological images of apoptosis marker, cleaved caspase-3, positive staining in male wild-type (*Pkd1*^+/+^) and *Pkd1^RC/RC^* mouse kidneys with *Atm^+/+^* or *Atm^+/−^* at 3 months of age. Scale bars, 50 µM.

To evaluate the long-term effect of reducing ATM on kidney cyst formation and growth *in vivo,* we next evaluated disease progression in *Pkd1^RC/RC^/Atm^+/−^* mice. At 3 months of age, *Pkd1^RC/RC^/Atm^+/−^* and *Pkd1^RC/RC^/Atm^−/−^* mice appeared healthy, and body weight was comparable to *Pkd1^RC/RC^/Atm^+/+^* mice (Figure 6). However, neither heterozygosity (*Atm^+/−^*) nor homozygosity (*Atm^−/−^*) for mutant *Atm* altered the progression of kidney enlargement or the percentage cyst area (Figure 6 and Figure 7). Similarly, no differences were also observed in these data when sub-analyzed by gender (Table S2). In addition, no changes in renal cell proliferation or interstitial fibrosis were detected in *Pkd1^RC/RC^/Atm^+/−^* mice compared to *Pkd1^RC/RC^/Atm^+/+^* mice (Figure 8). Furthermore, at 6 months of age, no differences in body weight, kidney or heart enlargement, cystic phenotype, or percentage cyst area were observed in *Pkd1^RC/RC^/Atm^+/−^* mice compared to historical control *Pkd1^RC/RC^/Atm^+/+^* mice (Figure S3). We also examined the downstream molecular effects of ATM loss by assessing the expression of γ-H2AX and p53 [51,52,53]. However, neither heterozygosity or homozygosity for *Atm* altered the expression of either of these proteins (Figure 9). Cleaved caspase-3 was expressed in the cytosol of the distal tubules in the kidney cortex of both wild-type and *Pkd1^RC/RC^* mice and in small to medium-sized cysts in *Pkd1^RC/RC^* mice (Figure 9E). However, qualitatively, no differences were detected with *Atm* heterozygosity (Figure 9E).

## 4. Discussion

Previously, we reported that DDR signaling was dysregulated in ADPKD [17], raising the possibility that modulating this pathway could be a novel approach to modify the progression of cystic kidney diseases [16]. The present study aimed to begin to evaluate this hypothesis and specifically investigate whether reducing either ATM or ATR attenuates kidney cyst growth. The results of this study revealed both expected and unexpected findings. First, inhibition of either ATM or ATR reduced cyst growth in vitro in the MDCK cyst model, but the latter was associated with a dysplastic cystic phenotype. Second, a clinically relevant ATM inhibitor, AZD0156, also reduced in vitro cyst growth using human ADPKD derived cells. Third, in keeping with these findings, AZD0156 reduced renal cell proliferation and augmented p53 expression in *Pkd1^RC/RC^* mice. Lastly, and unexpectedly, the chronic progression of cystic kidney disease in vivo was not altered by genetic ablation of ATM from birth, in either *Pkd1^RC/RC^/Atm^+/−^* or *Pkd1^RC/RC^/Atm^−/−^* mice. Taken together, these results, suggest that the ATM-DDR pathway is dispensable for the progression of cystic kidney disease in ADPKD.

Defective DDR signaling is a classical hallmark of cancer [54], and ADPKD has been coined as a “neoplasia in disguise” [55,56], sharing many of the oncogenic molecular abnormalities [7]. The DDR is a complex system consisting of ~450 proteins and each might be considered a potential target for therapy [57]. This study focused on evaluating the functional role of ATM by initially assessing the efficacy of three well-characterized pharmacological inhibitors (KU-60019, AZD1390, and AZD0156) on cyst growth in vitro [50]. KU-60019 is an ATP-competitive inhibitor that competently blocks ATM activity to disrupt DDR signaling and also suppresses AKT phosphorylation to attenuate cell survival [58]. Both AZD0156 and AZD1390 are highly selective, potent, and orally bioavailable ATM inhibitors developed for Phase 1 clinical trials [29,30,50]. Interestingly although AZD1390 is a more potent ATM inhibitor than AZD0156 (IC_50_ value of 0.78 vs. 0.58 nM) [29], we found that the latter had greater efficacy in reducing cyst growth in vitro. In any case, all three drug inhibitors consistently and potently reduced in vitro cyst growth in both the MDCK cells and also in a genetically orthologous human ADPKD cyst model using WT 9-12 cells. Moreover, the efficacy of AZD0156 on in vitro cyst growth was almost to the same magnitude as sirolimus, which is a well-known potent suppressor in vivo [59]. In our study, we also noted that the inhibition of ATM, depending on the specific drug and dosage, was also associated with reduced cell viability (as assessed by either the MTT assay or LDH leakage), and/or decreased cell proliferation (as assessed by BrdU incorporation assay), providing potential mechanisms by which cyst growth was reduced. Consistent with the in vitro studies, the short-term administration of AZD0156 in vivo in *Pkd1^RC/RC^* mice reduced renal cell proliferation and increased p53 expression, which is a driver of cell cycle arrest and apoptotic pathways [60]. Interestingly, p53 activation by AZD0156 was, however, not associated with an increase in cleaved caspase 3, suggesting that the caspase signal was not activated in this process.

Unexpectedly, despite these findings, the chronic progression of cystic kidney disease in vivo was not altered in either *Pkd1^RC/RC^/Atm^+/−^* or *Pkd1^RC/RC^/Atm^−/−^* mice and there were no changes in DNA damage, proliferation, apoptosis, or fibrosis in the kidneys. These disparate results suggest that ATM probably has a redundant role in the DDR pathway in ADPKD, and that mitotic catastrophe in cystic epithelial cells (as we had hypothesized would occur) was likely prevented by the activation of compensatory DDR pathways [61]. For example, DNA-dependent protein kinase (DNA-PK), another key PIKK protein involved in the DDR, is capable of phosphorylating ATM substrates and could potentially function in the absence of ATM [62,63]. Alternatively, a second possibility is that the different methods of reducing ATM have fundamental molecular differences, in that suppressing ATM kinase activity with pharmacological inhibitors is not equivalent to reduction or loss of ATM protein. For example, an ATM kinase-inactivating mutation is embryonically lethal in mice [64,65], whereas ATM protein null animals have less genomic instability than “kinase-dead” mutants and are viable [25]. Furthermore, in the presence of “kinase-dead” ATM or when kinase activity is inhibited pharmacologically, ATM is still recruited and engaged at the site of DNA damage but remains inactive, preventing other DDR proteins from taking over the function of DNA repair [61]. Thus, it would be of interest to investigate the long-term efficacy of ATM kinase inhibitors on cyst growth in preclinical models in future studies to verify the conclusions of the current study.

It is noteworthy that in our study, pharmacological inhibition of ATR with VE-821 reduced cyst growth in vitro, but concurrently produced dysplastic changes characterized by abnormal “hair-like” structures surrounding the spheroids, that was not observed with any of the ATM inhibitors. The cause of these changes and their significance is unknown, but we suspect that these phenotypic changes may suggest genome destabilization and be potential risk for mutagenesis in cystic kidney epithelial cells [66]. These conclusions are consistent with ATR being a more “essential” gene in the DDR than ATM [67]. In this regard, unlike ATM, mice homozygous for an ATR mutation are embryonically lethal, whereas heterozygous and hypo-morphic mice are viable [67]. Similar to our results, Hutcherson et al. reported that ATR kinase inhibitors (VE-821 and AZD6738) sensitize quiescent cells to cisplatin-induced death, but concurrently increase mutagenesis [68]. Due to these findings, in the present study, we did not further investigate the efficacy of ATR inhibitors *in vivo*, but clearly further experiments would be of interest to verify our in vitro data. In this regard, our results also raise broader questions regarding the long-term tolerability and feasibility of DDR inhibitors in the treatment of lifelong diseases, such as ADPKD, due to the potential for systemic side effects [67,69]. In this regard, the goal of the current paper was to first establish a functional role for ATM, and if positive then long-term strategies to reduce the risk of adverse effects will need consideration. These strategies might include intermittent dosing, use of inhibitors with lower toxicity, and/or low-dose combination therapy with other established disease-modifying drugs [70,71].

## 5. Conclusions

ADPKD is a fatal chronic disease with significant morbidity and limited therapeutic options for preventing kidney failure [6]. Current research on the role of the DDR pathway in ADPKD is very limited and, to our knowledge, this is the first study to investigate the efficacy of modulating ATM on experimental models. Our results showed that while ATM kinase inhibition reduced cyst growth in vitro, the reduction of ATM by genetic ablation did not alter the progression of cystic kidney disease in a well-established murine genetic ortholog of ADPKD. These data suggest that ATM has a dispensable role in kidney cyst formation and growth and/or that there are differing consequences between pharmacological ATM kinase inhibition compared to genetic ablation of ATM on disease outcomes in ADPKD. Further preclinical studies are needed to determine if other DDR pathways have a pathogenic role on cystic kidney disease progression in ADPKD.

## Figures and Tables

**Table 1:** Effect of VE-821 and KU-60019 on bromodeoxyuridine (BrdU) synthesis and lactate dehydrogenase (% LDH) leakage in Madin-Darby canine kidney (MDCK) cell monolayers after 24 h treatment.

Group	Dose	*n*	% BrdU Synthesis	% LDH Leakage
*Vehicle*		8	100.0 ± 2.5	23.5 ± 0.96
*VE-821*	2 µM	8	86.6 ± 8.2 *	22.0 ± 1.4
	5 µM	8	68.2 ± 8.3 *	21.7 ± 0.7
	10 µM	8	51.9 ± 4.6 *	22.6 ± 1.4
*KU-60019*	2 µM	8	86.1 ± 3.6 *	21.6 ± 1.0 *
	5 µM	8	65.0 ± 4.0 *	21.6 ± 1.34 *
	10 µM	8	13.5 ± 1.5 *	21.3 ± 0.8 *

Data expressed as mean + standard deviation; * *p* < 0.05 compared to vehicle.

## Data Availability

Data is contained within the article or supplementary material.

## Supplementary Materials

The following are available online at https://www.mdpi.com/2073-4409/10/3/532/s1, Figure S1: Representative melting peaks from genotyping for *Atm* to confirm presence of *Atm^+/+^*, *Atm^+/−^* or *Atm^−/−^*; Figure S2: Dysplastic hair-like outgrowths observed in three-dimensional MDCK in vitro cysts after treatment with pharmacological ATR inhibitor, VE-821; Figure S3: Body, kidney and heart weight and histological analysis of male *Pkd1^RC/RC^* mice with *Atm*^+/+^ or *Atm*^+/−^ at 6 months of age. Table S1: Percentage of dysplastic cysts following treatment with 2, 5 and 10 µM ATM inhibitor KU-60019 and ATR inhibitor VE-821. Table S2: Physical parameters and percentage cyst area of wild-type (*Pkd1^+/+^*) and *Pkd1*^RC/RC^ mice with *Atm^+/+^*, *Atm^+/−^*, or *Atm^−/−^* at 3 months of age, sub-analyzed by gender.

## References

[1] Groopman E.E., Marasa M., Cameron-Christie S., Petrovski S., Aggarwal V.S., Milo-Rasouly H., Li Y., Zhang J., Nestor J., Krithivasan P. (2019). Diagnostic Utility of Exome Sequencing for Kidney Disease. N. Engl. J. Med..

[2] Gall E.C.-L., Alam A., Perrone R.D. (2019). Autosomal dominant polycystic kidney disease. Lancet.

[3] Grantham J.J., Geiser J.L., Evan A.P. (1987). Cyst formation and growth in autosomal dominant polycystic kidney disease. Kidney Int..

[4] Ong A.C.M., Harris P.C. (2005). Molecular pathogenesis of ADPKD: The polycystin complex gets complex. Kidney Int..

[5] Rangan G.K., Tchan M.C., Tong A., Wong A.T.Y., Nankivell B.J. (2016). Recent advances in autosomal-dominant polycystic kidney disease. Intern. Med. J..

[6] Rangan G.K., Raghubanshi A., Chaitarvornkit A., Chandra A.N., Gardos R., Munt A., Read M.N., Saravanabavan S., Zhang J.Q., Wong A.T. (2020). Current and emerging treatment options to prevent renal failure due to autosomal dominant polycystic kidney disease. Expert Opin. Orphan Drugs.

[7] Seeger-Nukpezah T., Geynisman D.M., Nikonova A.S., Benzing T., Golemis E.A. (2015). The hallmarks of cancer: Relevance to the pathogenesis of polycystic kidney disease. Nat. Rev. Nephrol..

[8] Rowe I., Chiaravalli M., Mannella V., Ulisse V., Quilici G., Pema M., Song X.W., Xu H., Mari S., Qian F. (2013). Defective glucose metabolism in polycystic kidney disease identifies a new therapeutic strategy. Nat. Med..

[9] Battini L., Macip S., Fedorova E., Dikman S., Somlo S., Montagna C., Gusella G.L. (2008). Loss of polycystin-1 causes centrosome amplification and genomic instability. Hum. Mol. Genet..

[10] Li M., Qin S., Wang L., Zhou J. (2013). Genomic instability in patients with autosomal-dominant polycystic kidney disease. J. Int. Med. Res..

[11] Choi H.J.C., Lin J.-R., Vannier J.-B., Slaats G.G., Kile A.C., Paulsen R.D., Manning D.K., Beier D.R., Giles R.H., Boulton S.J. (2013). NEK8 Links the ATR-Regulated Replication Stress Response and S Phase CDK Activity to Renal Ciliopathies. Mol. Cell.

[12] Ta M.H.T., Schwensen K.G., Liuwantara D., Huso D.L., Watnick T., Rangan G.K. (2016). Constitutive renal Rel/nuclear factor-κB expression in Lewis polycystic kidney disease rats. World J. Nephrol..

[13] Cassini M.F., Kakade V.R., Kurtz E., Sulkowski P., Glazer P., Torres R., Somlo S., Cantley L.G. (2018). Mcp1 Promotes Macrophage-Dependent Cyst Expansion in Autosomal Dominant Polycystic Kidney Disease. J. Am. Soc. Nephrol..

[14] E Conduit S., Davies E.M., Ooms L.M., Gurung R., McGrath M.J., Hakim S., Cottle D.L., Smyth I.M., Dyson J.M., A. Mitchell C. (2019). AKT signaling promotes DNA damage accumulation and proliferation in polycystic kidney disease. Hum. Mol. Genet..

[15] Zhang J.Q.J., Burgess J., Stepanova D., Saravanabavan S., Wong A.T.Y., Kaldis P., Rangan G.K. (2020). Role of cyclin-dependent kinase 2 in the progression of mouse juvenile cystic kidney disease. Lab. Investig..

[16] Zhang J.Q.J., Saravanabavan S., Munt A., Wong A.T.Y., Harris D.C., Harris P.C., Wang Y., Rangan G.K. (2019). The role of DNA damage as a therapeutic target in autosomal dominant polycystic kidney disease. Expert Rev. Mol. Med..

[17] Zhang J.Q., Saravanabavan S., Chandra A.N., Munt A., Wong A.T., Harris P.C., Harris D.C., McKenzie P., Wang Y., Rangan G.K. (2021). Up-regulation of DNA Damage Response Signaling in Autosomal Dominant Polycystic Kidney Disease. Am. J. Pathol..

[18] Blackford A.N., Jackson S.P. (2017). ATM, ATR, and DNA-PK: The Trinity at the Heart of the DNA Damage Response. Mol. Cell.

[19] Lu X.-H., Mattis V.B., Wang N., Al-Ramahi I., Berg N.V.D., Fratantoni S.A., Waldvogel H., Greiner E., Osmand A., ElZein K. (2014). Targeting ATM ameliorates mutant Huntingtin toxicity in cell and animal models of Huntington’s disease. Sci. Transl. Med..

[20] Oberle C., Blattner C. (2010). Regulation of the DNA Damage Response to DSBs by Post-Translational Modifications. Curr. Genom..

[21] Giglia-Mari G., Zotter A., Vermeulen W. (2010). DNA Damage Response. Cold Spring Harb. Perspect. Biol..

[22] Awasthi P., Foiani M., Kumar A. (2015). ATM and ATR signaling at a glance. J. Cell Sci..

[23] Cortez D., Guntuku S., Qin J., Elledge S.J. (2001). ATR and ATRIP: Partners in Checkpoint Signaling. Science.

[24] Canman E.C. (2001). Replication checkpoint: Preventing mitotic catastrophe. Curr. Biol..

[25] Barlow C., Hirotsune S., Paylor R., Liyanage M., Eckhaus M., Collins F., Shiloh Y., Crawley J.N., Ried T., Tagle D. (1996). Atm-Deficient Mice: A Paradigm of Ataxia Telangiectasia. Cell.

[26] Menon V.R., Peterson E.J., Valerie K., Farrell N.P., Povirk L.F. (2013). Ligand modulation of a dinuclear platinum compound leads to mechanistic differences in cell cycle progression and arrest. Biochem. Pharmacol..

[27] Vecchio D., Daga A., Carra E., Marubbi D., Raso A., Mascelli S., Nozza P., Garrè M.L., Pitto F., Ravetti J.L. (2015). Pharmacokinetics, pharmacodynamics and efficacy on pediatric tumors of the glioma radiosensitizer KU60019. Int. J. Cancer.

[28] Smida M., De La Cruz F.F., Kerzendorfer C., Uras I.Z., Mair B., Mazouzi A., Suchankova T., Konopka T., Katz A.M., Paz K. (2016). MEK inhibitors block growth of lung tumours with mutations in ataxia–telangiectasia mutated. Nat. Commun..

[29] Durant S.T., Zheng L., Wang Y., Chen K., Zhang L., Zhang T., Yang Z., Riches L., Trinidad A.G., Fok J.H.L. (2018). The brain-penetrant clinical ATM inhibitor AZD1390 radiosensitizes and improves survival of preclinical brain tumor models. Sci. Adv..

[30] Riches L.C., Trinidad A.G., Hughes G., Jones G.N., Hughes A.M., Thomason A.G., Gavine P., Cui A., Ling S., Stott J. (2020). Pharmacology of the ATM inhibitor AZD0156: Potentiation of irradiation and olaparib responses pre-clinically. Mol. Cancer Ther..

[31] Dilley R.L., Verma P., Cho N.W., Winters H.D., Wondisford A.R., Greenberg R.A. (2016). Break-induced telomere synthesis underlies alternative telomere maintenance. Nat. Cell Biol..

[32] Ahuja A.K., Jodkowska K., Teloni F., Bizard A.H., Zellweger R., Herrador R., Ortega S., Hickson I.D., Altmeyer M., Mendez J. (2016). A short G1 phase imposes constitutive replication stress and fork remodelling in mouse embryonic stem cells. Nat. Commun..

[33] Gorthi A., Romero J.C., Loranc E., Cao L., Lawrence L.A., Goodale E., Iniguez A.B., Bernard X., Masamsetti V.P., Roston S. (2018). EWS–FLI1 increases transcription to cause R-loops and block BRCA1 repair in Ewing sarcoma. Nat. Cell Biol..

[34] Pike K.G., Barlaam B., Cadogan E., Campbell A., Chen Y., Colclough N., Davies N.L., De-Almeida C., Degorce S.L., Didelot M. (2018). The Identification of Potent, Selective, and Orally Available Inhibitors of Ataxia Telangiectasia Mutated (ATM) Kinase: The Discovery of AZD0156 (8-{6-[3-(Dimethylamino)propoxy]pyridin-3-yl}-3-methyl-1-(tetrahydro-2H-pyran-4-yl)-1,3-dihydro-2H-imidazo[4, 5-c]quinolin-2-one). J. Med. Chem..

[35] Ryan M.J., Johnson G., Kirk J., Fuerstenberg S.M., Zager R.A., Torok-Storb B. (1994). HK-2: An immortalized proximal tubule epithelial cell line from normal adult human kidney. Kidney Int..

[36] Loghman-Adham M., Nauli S.M., Soto C.E., Kariuki B., Zhou J. (2003). Immortalized epithelial cells from human autosomal dominant polycystic kidney cysts. Am. J. Physiol. Physiol..

[37] Nauli S.M., Rossetti S., Kolb R.J., Alenghat F.J., Consugar M.B., Harris P.C., Ingber N.E., Loghman-Adham M., Zhou J. (2006). Loss of Polycystin-1 in Human Cyst-Lining Epithelia Leads to Ciliary Dysfunction. J. Am. Soc. Nephrol..

[38] Yang B., Sonawane N.D., Zhao D., Somlo S., Verkman A.S. (2008). Small-Molecule CFTR Inhibitors Slow Cyst Growth in Polycystic Kidney Disease. J. Am. Soc. Nephrol..

[39] Turner C.M., King B.F., Srai K.S., Unwin R.J. (2007). Antagonism of endogenous putative P2Y receptors reduces the growth of MDCK-derived cysts cultured in vitro. Am. J. Physiol. Physiol..

[40] Holditch S.J., Brown C.N., Atwood D.J., Lombardi A.M., Nguyen K.N., Toll H.W., Hopp K., Edelstein C.L. (2019). A study of sirolimus and mTOR kinase inhibitor in a hypomorphic Pkd1 mouse model of autosomal dominant polycystic kidney disease. Am. J. Physiol. Physiol..

[41] Lannoy M., Valluru M.K., Chang L., Abdela-Ali F., Peters D.J., Streets A.J., Ong A.C. (2020). The positive effect of selective prostaglandin E2 receptor EP2 and EP4 blockade on cystogenesis in vitro is counteracted by increased kidney inflammation in vivo. Kidney Int..

[42] Mosmann T. (1983). Rapid colorimetric assay for cellular growth and survival: Application to proliferation and cytotoxicity assays. J. Immunol. Methods.

[43] Hopp K., Ward C.J., Hommerding C.J., Nasr S.H., Tuan H.-F., Gainullin V.G., Rossetti S., Torres V.E., Harris P.C. (2012). Functional polycystin-1 dosage governs autosomal dominant polycystic kidney disease severity. J. Clin. Investig..

[44] Genik P.C., Bielefeldt-Ohmann H., Liu X., Story M.D., Ding L., Bush J.M., Fallgren C.M., Weil M.M. (2014). Strain Background Determines Lymphoma Incidence in Atm Knockout Mice. Neoplasia.

[45] Hopp K., Hommerding C.J., Wang X., Ye H., Harris P.C., Torres V.E. (2014). Tolvaptan plus Pasireotide Shows Enhanced Efficacy in a PKD1 Model. J. Am. Soc. Nephrol..

[46] Hopp K., Wang X., Ye H., Irazabal M.V., Harris P.C., Torres V.E. (2015). Effects of hydration in rats and mice with polycystic kidney disease. Am. J. Physiol. Physiol..

[47] Gürtler A., Kunz N., Gomolka M., Hornhardt S., Friedl A.A., McDonald K., Kohn J.E., Posch A. (2013). Stain-Free technology as a normalization tool in Western blot analysis. Anal. Biochem..

[48] Taylor S.C., Berkelman T., Yadav G., Hammond M. (2013). A Defined Methodology for Reliable Quantification of Western Blot Data. Mol. Biotechnol..

[49] Charan J., Kantharia N.D. (2013). How to calculate sample size in animal studies?. J. Pharmacol. Pharmacother..

[50] Jin M.H., Oh D.-Y. (2019). ATM in DNA repair in cancer. Pharmacol. Ther..

[51] Banin S., Moyal L., Shieh S.-Y., Taya Y., Anderson C.W., Chessa L., Smorodinsky N.I., Prives C., Reiss Y., Shiloh Y. (1998). Enhanced Phosphorylation of p53 by ATM in Response to DNA Damage. Science.

[52] Canman C.E., Lim D.-S., Cimprich K.A., Taya Y., Tamai K., Sakaguchi K., Appella E., Kastan M.B., Siliciano J.D. (1998). Activation of the ATM Kinase by Ionizing Radiation and Phosphorylation of p53. Science.

[53] Burma S., Chen B.P., Murphy M., Kurimasa A., Chen D.J. (2001). ATM Phosphorylates Histone H2AX in Response to DNA Double-strand Breaks. J. Biol. Chem..

[54] Helleday T., Petermann E., Lundin C., Hodgson B., Sharma R.A. (2008). DNA repair pathways as targets for cancer therapy. Nat. Rev. Cancer.

[55] Grantham J.J. (1990). Polycystic Kidney Disease: Neoplasia in Disguise. Am. J. Kidney Dis..

[56] Harris P.C., Watson M.L. (1997). Autosomal dominant polycystic kidney disease: Neoplasia in disguise?. Nephrol. Dial. Transplant..

[57] Pearl L.H., Schierz A.C., Ward S.E., Al-Lazikani B., Pearl F.M.G. (2015). Therapeutic opportunities within the DNA damage response. Nat. Rev. Cancer.

[58] Golding S.E., Rosenberg E., Valerie N., Hussaini I., Frigerio M., Cockcroft X.F., Chong W.Y., Hummersone M., Rigoreau L., Menear K.A. (2009). Improved ATM kinase inhibitor KU-60019 radiosensitizes glioma cells, compromises insulin, AKT and ERK prosurvival signaling, and inhibits migration and invasion. Mol. Cancer Ther..

[59] Ta M.H.T., Schwensen K.G., Foster S., Korgaonkar M., Ozimek-Kulik J.E., Phillips J.K., Peduto A., Rangan G.K. (2016). Effects of TORC1 Inhibition during the Early and Established Phases of Polycystic Kidney Disease. PLoS ONE.

[60] Kurbegovic A., Trudel M. (2020). The master regulators Myc and p53 cellular signaling and functions in polycystic kidney disease. Cell Signal..

[61] Menolfi D., Zha S. (2020). ATM, ATR and DNA-PKcs kinases—the lessons from the mouse models: Inhibition ≠ deletion. Cell Biosci..

[62] Callén E., Jankovic M., Wong N., Zha S., Chen H.-T., Difilippantonio S., Di Virgilio M., Heidkamp G., Alt F.W., Nussenzweig A. (2009). Essential Role for DNA-PKcs in DNA Double-Strand Break Repair and Apoptosis in ATM-Deficient Lymphocytes. Mol. Cell.

[63] Stiff T., O’Driscoll M., Rief N., Iwabuchi K., Löbrich M., Jeggo P.A. (2004). ATM and DNA-PK Function Redundantly to Phosphorylate H2AX after Exposure to Ionizing Radiation. Cancer Res..

[64] Daniel J.A., Pellegrini M., Lee B.-S., Guo Z., Filsuf D., Belkina N.V., You Z., Paull T.T., Sleckman B.P., Feigenbaum L. (2012). Loss of ATM kinase activity leads to embryonic lethality in mice. J. Cell Biol..

[65] Yamamoto K., Wang Y., Jiang W., Liu X., Dubois R.L., Lin C.-S., Ludwig T., Bakkenist C.J., Zha S. (2012). Kinase-dead ATM protein causes genomic instability and early embryonic lethality in mice. J. Cell Biol..

[66] Karnitz L.M., Zou L. (2015). Molecular Pathways: Targeting ATR in Cancer Therapy. Clin. Cancer Res..

[67] Weber A.M., Ryan A.J. (2015). ATM and ATR as therapeutic targets in cancer. Pharmacol. Ther..

[68] Hutcherson R.J., Kemp M.G. (2019). ATR kinase inhibition sensitizes quiescent human cells to the lethal effects of cisplatin but increases mutagenesis. Mutat. Res. Mol. Mech. Mutagen..

[69] Bradbury A., Hall S., Curtin N., Drew Y. (2020). Targeting ATR as Cancer Therapy: A new era for synthetic lethality and synergistic combinations?. Pharmacol. Ther..

[70] Sans-Atxer L., Joly D. (2018). Tolvaptan in the treatment of autosomal dominant polycystic kidney disease: Patient selection and special considerations. Int. J. Nephrol. Renov. Dis..

[71] Kou P., Wei S., Xiong F. (2019). Recent Advances of mTOR Inhibitors Use in Autosomal Dominant Polycystic Kidney Disease: Is the Road Still Open?. Curr. Med. Chem..

