# Supplementary material for: Effect of Reducing Ataxia-Telangiectasia Mutated (ATM) in Experimental Autosomal Dominant Polycystic Kidney Disease
Source: Cells. 2021 Mar 3;10(3):532. doi: 10.3390/cells10030532 (PMC8000896; doi:10.3390/cells10030532)
Supplement: Supplementary file 1 [file cells-10-00532-s001.pdf]

**Figure S1**

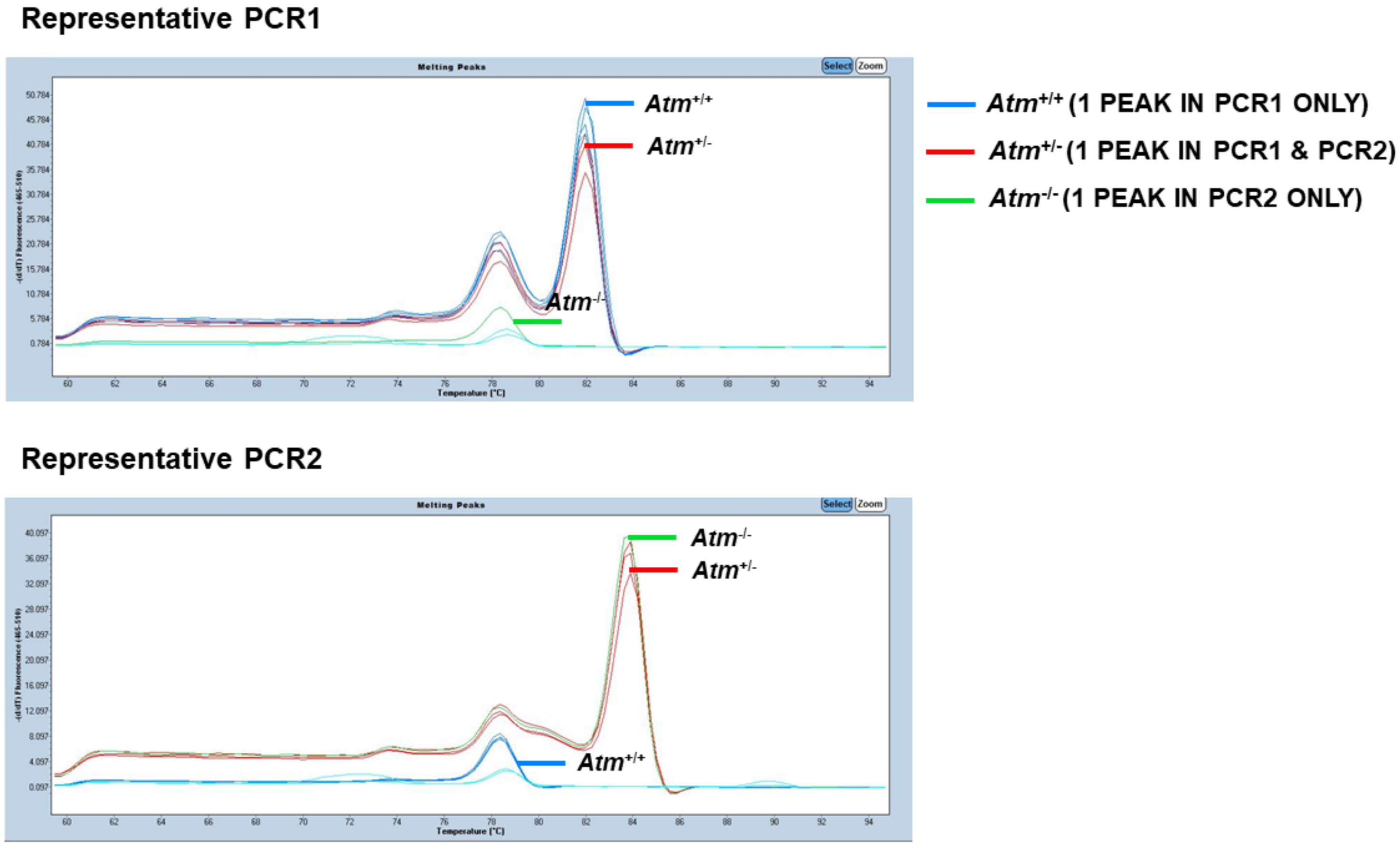


**Figure S1. Representative melting peaks from genotyping for *Atm* to confirm presence of *Atm*$^{+/+}$, *Atm*$^{+/-}$ or *Atm*$^{-/-}$.**

A two-step polymerase chain reaction (PCR) was used to confirm genotyping where one peak in PCR1 only indicated *Atm*$^{+/+}$, a peak in both PCR1 and PCR2 indicated *Atm*$^{+/-}$, and one peak in PCR2 only indicated *Atm*$^{-/-}$.

**Figure S2**

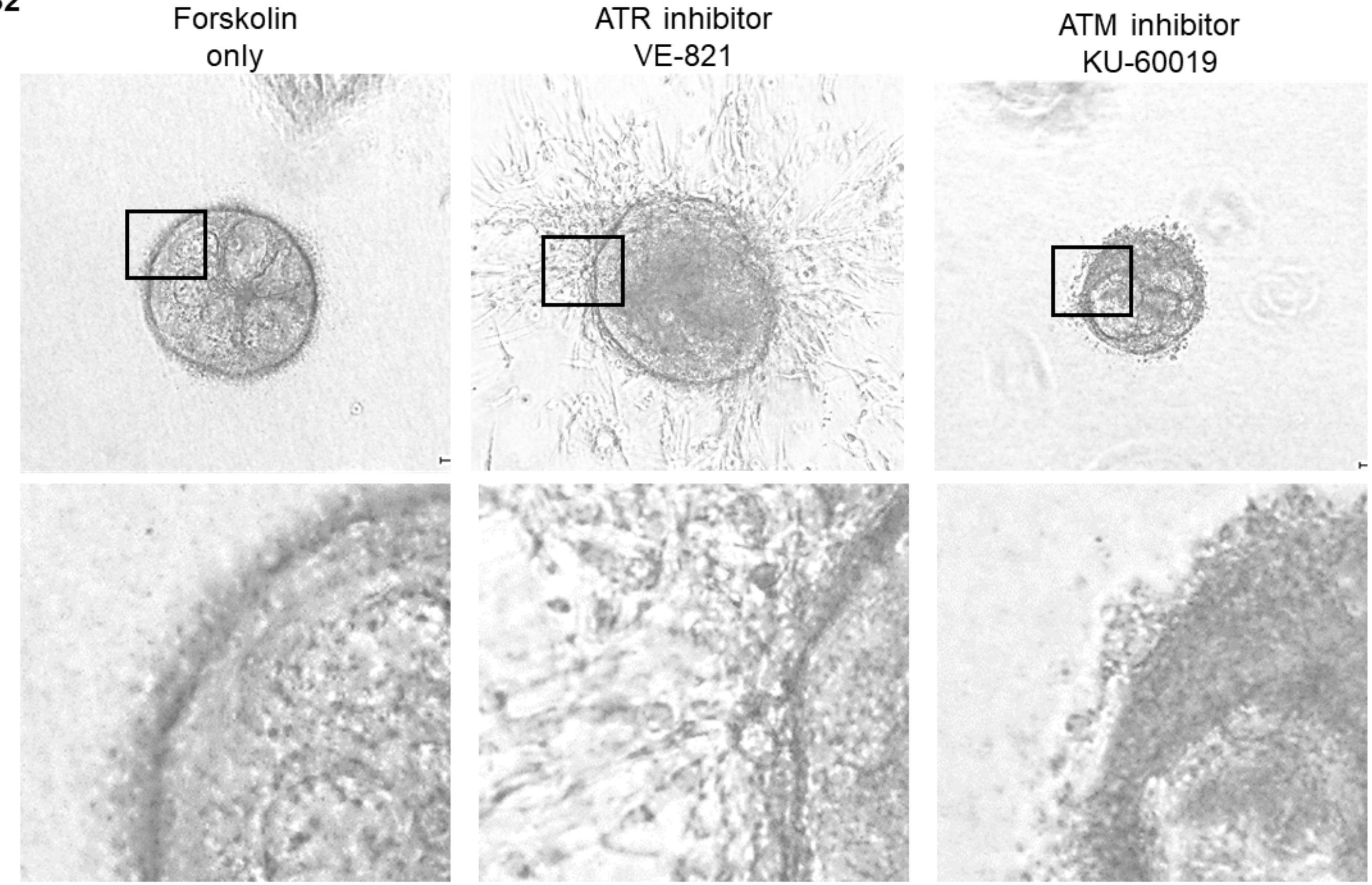


**Figure S2.** Dysplastic hair-like outgrowths observed in three-dimensional MDCK *in vitro* cysts after treatment with pharmacological ATR inhibitor, VE-821 are distinct from those observed after treatment with pharmacological ATM inhibitor KU60019 or vehicle.

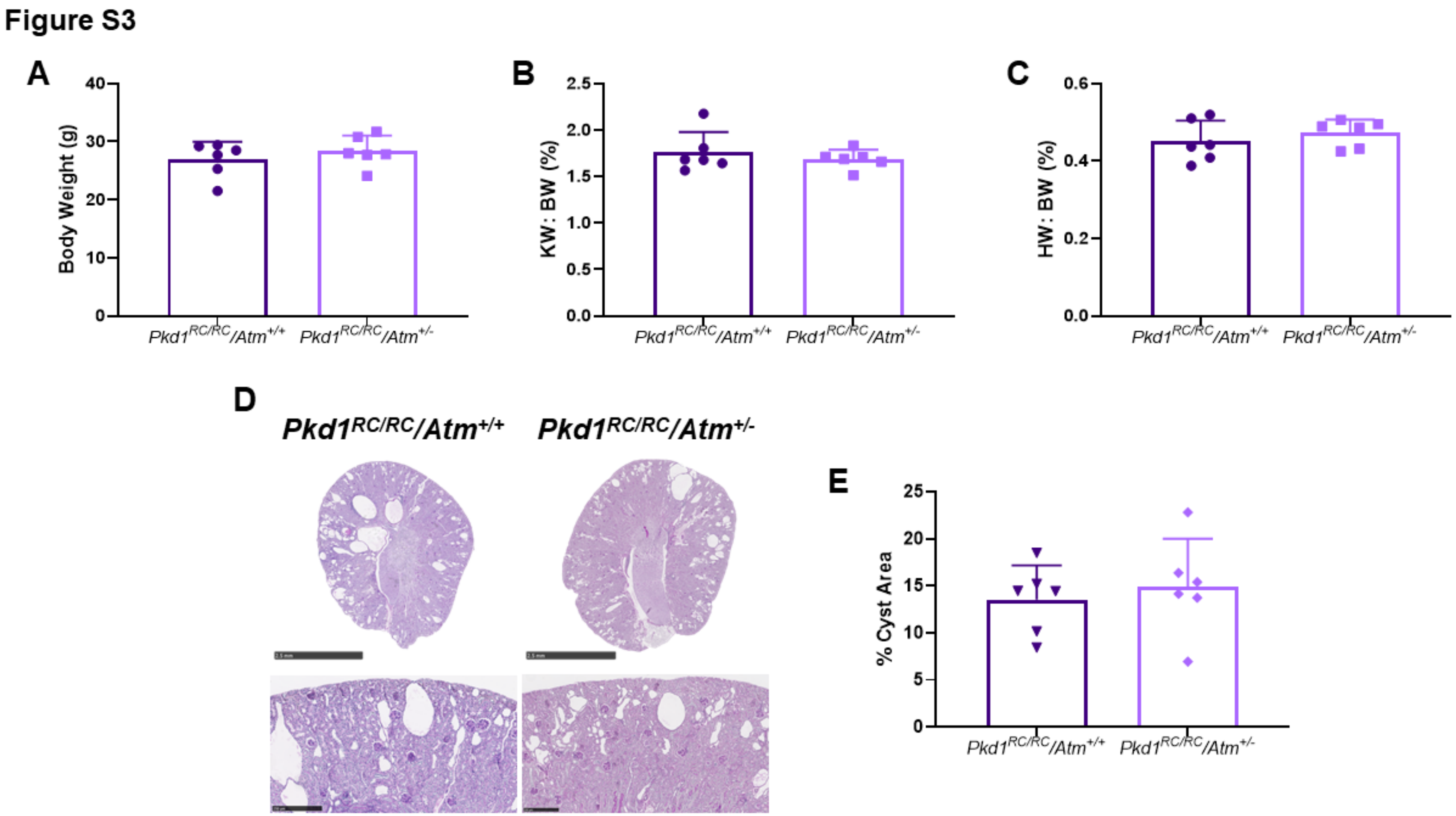


**Figure S3. Body, kidney and heart weight and histological analysis of male *Pkd1*$^{RC/RC}$ mice with *ATM*$^{+/+}$ or *ATM*$^{+/-}$ at 6 months of age.** (A) Body weight. (B) Two kidney weight to body weight ratio (KW: BW). (C) Heart weight to body weight ratio. (D) Representative whole-slide digital images of Periodic Acid Schiff (PAS)-stained kidney sections. (E) Percentage cyst area. Statistical analyses were performed by independent t-test. Data presented as means ± SD (n=6 per group).

**Table S1. Percentage of dysplastic cysts following treatment with 2, 5 and 10 μM ATM inhibitor KU-60019 and ATR inhibitor VE-821. Dysplastic cysts were defined as cysts with extensive, long 'hair-like' growth radiating from membrane of cyst.**

| | | Total number of cysts counted | Dysplastic cysts* | % Dysplastic cysts |
|---|---|---|---|---|
| **KU60019** | **2 μM** | 46 | 0 | 0.0 |
| | **5 μM** | 40 | 1 | 2.5 |
| | **10 μM** | 47 | 0 | 0.0 |
| **VE821** | **2 μM** | 46 | 12 | 26.1 |
| | **5 μM** | 46 | 7 | 15.2 |
| | **10 μM** | 40 | 0 | 0.0 |

**Table S2.** *Physical parameters and percentage cyst area of wild-type (Pkd1$^{+/+}$) and Pkd1$^{RC/RC}$ mice with Atm$^{+/+}$, Atm$^{+/-}$ or Atm$^{-/-}$ at 3 months of age, sub-analyzed by gender.*

| Group | Gender | n | Body Weight (g) | KW: BW (%) | HW: BW (%) | Cyst Area (%) |
|---|---|---|---|---|---|---|
| *Pkd1$^{+/+}$/Atm$^{+/+}$* | M | 4 | 27 ± 0 | 1.02 ± 0.04 | 0.48 ± 0.05 | 3.7 ± 1.7 |
| | F | 4 | 20 ± 1* | 1.00 ± 0.01 | 0.50 ± 0.02 | 5.7 ± 3.6 |
| *Pkd1$^{+/+}$/Atm$^{+/-}$* | M | 4 | 26 ± 1 | 1.09 ± 0.09 | 0.49 ± 0.06 | 4.6 ± 1.4 |
| | F | 4 | 19 ± 1* | 0.97 ± 0.07 | 0.53 ± 0.03 | 5.9 ± 4.2 |
| *Pkd1$^{+/+}$/Atm$^{-/-}$* | M | 2 | 23 ± 1 | 1.03 ± 0.02 | 0.47 ± 0.01 | 4.7 ± 2.2 |
| | F | 1 | 18 | 1.04 | 0.52 | 4.5 |
| *Pkd1$^{RC/RC}$/Atm$^{+/+}$* | M | 10 | 23 ± 1# | 1.67 ± 0.11# | 0.49 ± 0.06 | 16.6 ± 5.4# |
| | F | 10 | 19 ± 1* | 1.46 ± 0.21# | 0.49 ± 0.06 | 15.3 ± 6.0# |
| *Pkd1$^{RC/RC}$/Atm$^{+/-}$* | M | 10 | 24 ± 3 | 1.74 ± 0.22# | 0.49 ± 0.04 | 17.7 ± 4.9# |
| | F | 10 | 19 ± 1* | 1.54 ± 0.17# | 0.47 ± 0.04 | 16.8 ± 3.5# |
| *Pkd1$^{RC/RC}$/Atm$^{-/-}$* | M | 8 | 20 ± 2 | 1.63 ± 0.18# | 0.47 ± 0.05 | 14.1 ± 4.7 |
| | F | 2 | 17 ± 0 | 1.27 ± 0.08 | 0.52 ± 0.03 | 7.6 ± 0.3 |

KW: BW, two kidney weight to body weight ratio; HW:BW, heart weight to body weight ratio.

*P<0.05 compared to type-matched males and #P<0.05 compared to gender, *Atm*-matched *Pkd1$^{+/+}$* by one-way ANOVA, followed by post-hoc analysis with the Tukey Kramer HSD test. Data presented as means ± SD.
